# The Correlation between Serum ApoA1 and B and Coronary Artery Disease as Well as Its Severity

**Published:** 2014-01-01

**Authors:** Navid Reza Mashayekhi, Saeid Sadrnia, Ali Chehrei, Javad Javaheri

**Affiliations:** 1 Amirkabir Hospital, Arak University of Medical Sciences, Arak, IR Iran; 2 Thyroid Research Center, Arak University of Medical Sciences, Arak, IR Iran; 3 Medical School, Community Medicine Department, Arak University of Medical Sciences, Arak, IR Iran

**Keywords:** Apolipoprotein A-I, Apolipoproteins B, Coronary Artery Disease, Atherosclerosis

## Abstract

**Background:**

Some patients with Coronary Artery Disease (CAD) have no well-known risk factors of this disease, but are diagnosed with cardiovascular events. The present study aimed to assess the association between Apo A1 and ApoB and the severity of CAD and determine whether these parameters are better predictors of Ischemic Heart Disease (IHD).

**Methods:**

In this case control study, 271 individuals who were suspicious of having CAD and had been referred to Arak Amir-al-Momenin hospital underwent coronary angiography. Based on the results of angiography, the participants with presence or absence of coronary artery stenosis were allocated into the case and the control group, respectively. The severity of CAD involvement was determined by Gensini score. The data were entered into the SPSS statistical software and analyzed through parametric and non-parametric tests, sensitivity analysis, and logistic regression.

**Results:**

The results revealed no significant correlation between apoA-1 and severity of CAD involvement (GS) (r = 0.017, P = 0.797). However, a significant correlation was found between apoB and GS (r = 0.127, P = 0.047). Logistic regression model showed ApoB, sex, DM and, FH as the only proper predictors of IHD (P < 0.048, P < 0.002, P < 0.040, and P < 0.001, respectively). In comparison to angiography for diagnosis of CAD, ROC analysis represented ApoB as a more useful predictor (P = 0.023).

**Conclusions:**

In addition to measurement of conventional parameters for assessing CAD high risk groups, according to the results of this study using ApoB would be resonable as well. Further investigations are recommended to clear the problem.

## 1. Background

Cardiovascular diseases are prevalent around the world as well as in Iran. Among these diseases, atherosclerosis is one of the major causes of death worldwide (1). Knowing the risk factors of atherosclerosis plays a significant role in prevention and treatment of this disease. Today, in addition to the known risk factors, other new risk factors, including APO lipoprotein A1 and APO lipoprotein B, have been proposed for this disease (2). In clinical conditions, High Density Lipoprotein Cholesterol (HDL–C) and APO A1 have anti-atherogenic properties. APO lipoprotein B as well as the potentially atherogenic cholesterols plays a role, as well. In comparison to Low-Density Lipoprotein Cholesterol (LDL–C), the APO B / APO A1 ratio is a better indicator of the risk of coronary heart disease (3). The results of a study which was conducted on a group of men at Harvard medical school also showed that compared to the cholesterols which are carried by lipoproteins and measured by non HDL-C, the plasma concentration of the atherogenic lipoprotein particles which are measured by APO B work better in predicting Coronary Artery Disease (CAD) (4).

Moreover, Sabino et al. in 2008 showed that after controlling the effect of variables, such as age, sex, smoking, hypertension, and dyslipidemia, APO B and APO B / APO A1 ratio were independently associated with brain stroke and peripheral vascular disease in young adults (5). Besides, the study by Ray et al. in 2009 indicated that APO B / APO A1 ratio, similar to TG / HDL ratio, gave the same prognostic information in a patient with acute coronary syndrome who was under treatment with statin (6).

Sweetnom et al. in a prospective study showed a strong relationship between the level of APO lipoprotein B and the incidence of Ischemic Heart Disease (IHD). However, no such relationship was found after adjustment for total cholesterol level. Similarly, a strong correlation was observed between the low level of APO lipoprotein A 1 and the incidence of IHD. However, this correlation disappeared when the HDL level was added to the model (7). Nevertheless, more studies are required in order to determine whether APO A1 and APO B have clear superiority over the traditional lipid measurements as the predictors of cardiovascular disease risk (8).

Considering the above-mentioned contradictions, it is necessary to perform more investigations, especially in the Iranian community. Hence, the present study aims to assess the relationship between APO lipoproteins and the severity of CAD.

## 2. Materials and Methods

This case control study was conducted on all the individuals who were suspected for having CAD and had been admitted to the angiography section of Amir-al-Momenin hospital, Arak, Iran. The demographic data, medical history of CAD, history of coronary angiography / angioplasty, hypertension, diabetes mellitus, and smoking, and family history of CAD in the first degree relatives of the patients were collected through a previously designed researcher-made questionnaire. The inclusion criterion of the study was referring to the heart angiography section of Amir-al-Momenin hospital, Arak, Iran. On the other hand, the exclusion criteria of the study were having the history of chronic renal failure, being under treatment by blood lipid lowering drugs (statins, fibrate, and nicotinic acids), and being under treatment by sex hormones (androgens, estrogens, and progesterone). Based on the results of angiography, the participants with presence or absence of coronary artery stenosis were allocated into the case and the control group, respectively.

Finally, 271 patients were enrolled into the current study. After providing the participants with sufficient explanation and obtaining written informed consents from all the patients before angiography, blood samples were taken. The patients were also informed about the safety and free cost of blood sampling. All the blood samples were sent to laboratory for measurement of serum levels of lipoprotein A1, lipoprotein B, cholesterol, triglyceride, LDL, HDL, VLDL, and Fasting Blood Sugar (FBS). Lipid profile was measured using Hitachi 911 auto analyzer which had the confirmation of Food and Drug Administration (FDA). In addition, quality control was done in normal and high levels in each series. Besides, LDL was measured by the direct method. Furthermore, Minireph device, which has the capability to measure the indicators by the nephlometry method, was used to measure APO A and APO B. Then, the patients underwent angiography using Siemens angiography device (Axiom senses). The results of angiography were reviewed by two cardiologists who did not have any information about the patients' medical history. It should be noted that the patients' angiography movies were interpreted by the two cardiologists independently.

The severity of CAD was determined by Gensini criteria and defined by Gensini Score (GS) (1). After all, the data were entered into the SPSS statistical software and analyzed using descriptive statistics and parametric as well as non-parametric statistical tests, including independent T-test, Mann-Whitney test, Spearman's correlation test, sensitivity analysis, and logistic regression. Besides, significance level was set at 0.05 (type 1 error). Except for total cholesterol, other variables did not have normal distribution; therefore, non-parametric tests were employed. Of course, no difference was observed between the parametric and non-parametric tests results. Thus, we announced the results of the parametric tests.

## 3. Results

A total of 271 patients participated in the present study. After angiography, 160 participants (59%) were diagnosed with CAD and were consequently allocated to the case group. On the other hand, 111 subjects (41%) did not have CAD and were considered as the control group. The mean age of the study participants was 60.63 ± 12.5 years. Besides, the mean age of the CAD and the control group was 61.02 ± 11.06 and 60.7 ± 12.3 years, respectively and the difference was not statistically significant (P = 0.855).

Table 1 shows the means and standard deviations of FBS, APO B, APO A1, VLDL- C, HDL- C, LDL- C, total cholesterol, and TG in CAD and non-CAD groups. As the table depicts, no significant difference was observed between the patients and the control group regarding FBS, TG, total cholesterol, LDL- C, HDL- C, VLDL, and APO A1. However, the results of independent T-test indicated a significant difference between the two groups concerning the mean of APO B (P = 0.023).

**Table 1.** Comparison of Fasting Blood Sugar, Blood Lipids Profile, and Lipoproteins A1 and B in the Patients and the Control Group^*^

Group	Non-CAD (n = 111)	CAD (n = 160)	P value
	SD ± Mean	SD ± Mean	
**FBS**	111.890 ± 54.359	114.560 ± 52.959	0.6
**TG**	140.960 ± 104.437	162.270 ± 97.185	0.1
**CHOL**	187.362 ± 39.875	192.432 ± 50.674	0.4
**LDL**	105.361 ± 29.671	120.488 ± 159.176	0.3
**HDL**	45.846 ± 15.171	44.081 ± 15.174	0.1
**VLDL**	29.473 ± 24.676	34.736 ± 35.916	0.3
**APOA1**	128.741 ± 171.043	123.371 ± 145.253	0.897
**APOB**	109.695 ± 173.136	110.019 ± 108.255	0.023

^*^Significant at < 0.05

In this study, Spearman's test was used to assess the correlation between APO A1 and APO B and the severity of coronary artery stenosis measured by GS score. According to the results, no significant correlation was observed between GS and APO A1 (r = 0.017, P = 0.797), while GS score and APO B were correlated significantly (P = 0.047, r = 0.127).

Table 2 shows the ROC curve analysis results for APO A1, APO B, and APO B / APO A1 ratio. In this analysis, indicators, such as Area Under Curve (AUC), CI: 95%, P value, cut-off point, sensitivity, and specificity, were described for the above variables. In comparison to angiography for diagnosis of coronary artery disease, by measuring APO B level as an alternative test, the coverage under ROC curve was 58.5%. In fact, AUC was only significant for APO B (P < 0.023). This implies that using APO B test might be useful for diagnosing CAD, but this is not the case for APO A1 and APO B / APO A1 ratio. In other words, APO A1 or APO B / APO A1 ratio cannot be used for diagnosis of CAD with certainty.

**Table 2.** ROC Curve Analysis Results for APO A1, APO B, and APO B / APO A1 Ratio

Indicators	Area Under Curve(AUC)	CI: 95%	P value	Cut off point	Sensitivity	Specificity
**APOB**	58.5	51.3 - 65.7	0.023 ^*^	89.5	59.3	59.4
**APO A1**	50.5	43.1 - 57.8	0.897	99.5	51	49.5
**APO B / APO A1**	54.3	46.9 - 61.8	0.249	0.9	51	50.5

^*^Significant at < 0.05

**Figure 1.**
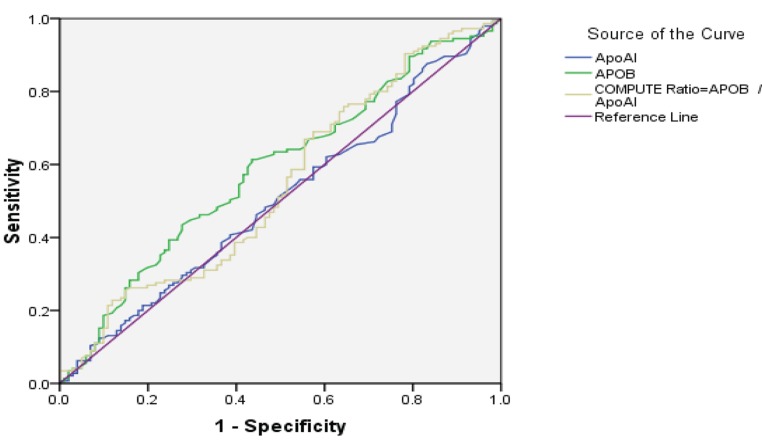
Roc Curve Analysis for APO A1, APO B, and APO B / APO A1 Ratio

It should be noted that to detect the best cut off point for APO B with maximum sensitivity and specificity, coordinates of the ROC curve tables must be used. Examining this table exactly showed that the cut-off point of 89.5 had such a feature. Figure 1. illustrates ROC Curve Analysis for APO A1, APO B, and APO B / APO A1 ratio.

To predict the presence or absence of CAD (dependent variable), logistic regression analysis was used. The variables entered into the model (covariates) included sex, age, history of hypertension (HTN), history of diabetes (DM), family history of premature CAD (FH), cholesterol, triglyceride, HDL, LDL, VLDL, APO B, APO A1, and APO B / APO A1 ratio. Among these variables, only APOB, sex, DM, and FH were identified as the predictor variables. The covariates were analyzed through the “Entered method”. The whole prediction power of the model was 27.8% for all the variables (Nagelkerk R square). Moreover, the probability value of Hosmer-Lemeshow goodness of fit test was not significant (P < 0.072). This implies that the entered data were fitted with the model. On the basis of the classification table results, the correct prediction percents for diagnosing the patients (sensitivity) and the healthy individuals (specificity) were 52.2% and 80.5%, respectively. This result indicated that the overall accuracy of the model was 68.3%. (Table 3) 

**Table 3.** Logistic Regression for Predicting Coronary Artery Disease according to APO B, APO A1, APO B / APO A1 Ratio, Sex, Family History, and Diabetes (the Dependent Variable Was the Presence or Absence of Coronary Artery Stenosis)

Variable	Odds Ratio (OR)	P value	CI: 95%	
			Lower	Upper
**APO B**	1.039	0.048 ^*^	1.001	1.078
**APO A1**	0.992	0.516	0.969	1.016
**APO B / APO A1**	0.283	0.475	0.009	9.002
**Sex**	3.664	0.002 ^*^	1.610	8.339
FH ^**^	3.629	0.001 ^*^	1.685	7.818
DM ^***^	4.172	0.040 ^*^	1.070	16.271

^*^Significant at < 0.05, ** Family History, *** Diabetes Mellitus

## 4. Discussion

The results of the present study showed that among gender, age, cholesterol, triglycerides, HDL, LDL, APO A1, APO B, and the APO B / APO A1 ratio, just APo B, FH, DM and gender were the predictors of CAD. In contrast to angiography method for diagnosis of CAD, measurement of APO B level can be useful as a laboratory test. In addition, the best cut off point for APO B was 89.5. However, measuring APO A1 and APO B / APO A1 ratio cannot be useful in laboratory tests.

The findings of the current study indicated no significant correlation between the LDL level and CAD. This might be due to the fact that measurement of LDL only shows the total cholesterol mass in LDL particles, but does not give any information about the number and size of the LDL particles. The size of the LDL particles has an inverse effect on their ability to cross through the arterial endothelium, penetration to intima, and risk of atherosclerosis. Nevertheless, the results showed a significant correlation between the APO B level and the severity of CAD. This might result from the fact that atherogen particles in plasma, such as LDL, HDL, and VLDL, contain APO B molecules. Indeed, APO B directly reflects the number of plasma atherogenic lipoproteins since each particle of LDL, IDL, and VLDL involves just one APO B. On the other hand, no significant correlation was observed between APO A1 and the severity of CAD.

The results of the present study are in agreement with those of the study Pischon et al. conducted in Harvard University, demonstrating that APO B could predict the occurrence of CAD (4).

Similar results were also obtained by Sabin et al. They found that after adjusting the role of gender, age, smoking, and HTN, APO B level and APO B / APO A1 ratio were independently correlated to peripheral atherosclerosis and brain stroke (5).

In a prospective study by Sweetnam et al., after adjusting the results for cardiovascular risk factors, regardless of plasma lipids, a strong correlation was found between the incidence of IHD and high level of APO lipoprotein B as well as low level of APO lipoprotein A1. However, this trend disappeared after entering the total cholesterol size into the model. These results are inconsistent with our findings because by controlling the other variables predictor power of ApoB remained constant (7).

The results of a study which was performed by Yazici et al. in Turkey revealed a significant correlation between the APO A1 serum level and cardiac troponins in the patients with angina pectoris. However, no significant relationship was found between the severity of CAD and the number of diseased vessels. These results are in line with our study findings regarding the correlation between APO A1 and the severity of CAD stenosis.

In another study which was carried out by Habib et al. on 140 patients in Saudi Arabia, a significant correlation was observed between APO A1 and severity and extension of coronary arteries stenosis in the patients but not in the control group (9). However, no such result was obtained in our study. In another study conducted by Dirisamer et al., LDL level, APO B level, and APO B / APO A1 ratio were significantly associated with the family history of IHD in the children with the family history of this disease. In the children whose fathers had the history of infraction, however, only APO B showed the highest correlation with the family history (10). These results are nearly similar to those of the present study.

According to the results of this study, plasma APO B test could be added to the common plasma lipid profile in laboratory tests in the individuals who are at risk of CAD. If the results of APO B laboratory test are positive, this factor should be considered as a risk factor for CAD.
